# Consumer response to media information: the case of grapefruit-medicine interaction

**DOI:** 10.1186/s13561-015-0069-z

**Published:** 2015-10-26

**Authors:** Hyeyoung Kim, Lisa A. House, Matthew Salois

**Affiliations:** Food and Resource Economics Department, University of Florida, Gainesville, USA

**Keywords:** Food-medicine interaction, Sample selection, Grapefruit, Interaction terms, Non-linear model, Q13, I12, L82

## Abstract

This study measured the effect of media exposure on grapefruit/grapefruit juice consumption changes, in particular grapefruit-medicine interaction. Respondents’ attitudes about health news on television and the internet were measured to account for consumers exposed versus not exposed to such information. Results of a sample selection model show that consumer attitudes toward health news were significantly related to exposure to media information. Also, news exposure about grapefruit-medicine interaction has a tendency to result in reduced grapefruit consumption. Consumers who are directly affected by the medication interaction significantly react to the news, and the effect varies by age. Even though consumer’s age was positively related to the probability of increased grapefruit consumption, when consumers took the medication, consumer’s age was negatively related to the probability of increased grapefruit consumption.

## Background

There is an extensive literature examining the role of media information and coverage on influencing consumer perception and behavior, particularly with respect to food choices. The relationship between media exposure and changes in consumer attitudes, opinions, and choices has been examined in a number of areas, including food safety/contamination [1, 2], nutrition and health [3, 4], animal welfare [5, 6], and biotech foods [7, 8]. Consumer response to media information on food-medicine interactions has yet to be examined.

Interactions between medicine intake and nutrient intake from food consumption can result in unintentional side-effects involving treatment failure or toxicity. Most food-medicine interactions are caused by changes in the bioavailability of the medicine and can lead to lengthened treatment time, hospitalization, increased morbidity, and in some cases mortality [9]. Although a number of food-medicine interactions have been documented in the medical literature, interactions with grapefruit and grapefruit juice have received considerable attention [10]. In addition to the professional medical literature, the general media continues to report on grapefruit-medicine interaction with various degrees of scientific accuracy [11]. Regardless of the accuracy, such information may lead to consumption changes.

The goal of this research is to measure the effect of media exposure on grapefruit/grapefruit juice consumption, with particular attention on news relating to grapefruit-medicine interaction. A survey is employed to understand respondents’ attitudes about health news on television and the internet and to measure the effect of media coverage on consumption changes of grapefruit and grapefruit juice. Despite the growth of internet use, traditional media such as television and newspapers are still important. Over 90 % of Americans continue to receive their news from television, internet, and newspapers; health and medicine news are commonly searched for along with weather and national events [12]. Due to online accessibility of newspapers, we focused on television and the internet as channels of media exposures.

To measure consumer response to media information, we divided media content into two factors: exposure to the 2012 grapefruit health news release and the impact the news had on those consumers’ grapefruit consumption. First, this study focuses on understanding media exposure to health news, with the concept that different characteristics might lead one individual to be more aware of health news through television or the internet than through other outlets. Second, this study explores the effect of news media on consumer purchases of grapefruit/grapefruit juice, focusing on those consumers who had been exposed to the news only (i.e., media-exposed consumers). Furthermore, this study compares the effects based on subjects’ age, as it is an important characteristic explaining consumption patterns of grapefruit/grapefruit juice [13]. We also expect that age may be a critical factor determining use of prescription medications.

Estimating the effect of media information on choice and behavior must take into account consumers who are exposed versus not exposed to such information. As such, this article uses a sample selection model, similar to a model by Heckman [14], modified to account for ordered responses. In particular, a binary probit equation is used to assess for media exposure (selection equation) and an ordered probit equation is used to assess changes in grapefruit/grapefruit juice consumption (outcome equation).

### Literature review

Grapefruit and grapefruit juice are widely consumed for their positive health benefits. Grapefruit has been found to have a preventative influence on a number of chronic diseases, including cardiovascular disease and cancer [11]. Fujioka et al. [15] found that a consumer group that consumed half of a fresh grapefruit before every meal for 12 weeks showed significant weight loss and improved insulin resistance. Dow et al. [16], on the other hand, did not find a significant relationship between grapefruit consumption and weight loss, but they did find improvements in blood pressure and lipids among a treatment group in which participants ate half of a fresh grapefruit with each meal for six weeks. Staudte, Sigusch, and Glockmann [17] found that the intake of grapefruit leads to an increase in plasma vitamin C level and improves sulcus bleeding scores in periodontitis patients. There is a sizeable literature on such benefits, both in the medical profession and in the general media.

Concurrent with the literature and media on the positive benefits of consuming grapefruit and grapefruit-containing products, there is also information on the negative issues associated with consumption of grapefruit, predominately on grapefruit-medicine interactions. Food-medicine interactions are not uncommon, and there are hundreds of foods, including broccoli, coffee, and dairy products, that have the potential to interact with certain medicines [9, 18]. Potential grapefruit-medicine interaction was initially discovered and reported in 1991 by Bailey et al. [19], which has received considerable attention in both the medical literature and the general media. Particular focus has been given to statins, lipid-lowering medicines, antibiotics, and calcium channel blockers [20].

A study by Bailey, Dresser, and Arnold [21] found that more than 85 medicines have the possibility of interacting with grapefruit, and of these medicines, 43 have interactions that can result in serious adverse effects. In addition, they indicated that older patients have the greatest possibility of ingesting grapefruit with interacting medications and are the most vulnerable to adverse clinical consequences. This study was initially released on 26 November 2012 and was widely reported in the popular media, including spots on major networks. ABC, CBS, NBC, and Fox all cited the study in news stories about grapefruit-medication interaction on 26 and 27 November 2012. The news triggered public interest. Based on searching the news from ProQuest® between 26 November 2012 and 3 January 2013, the publication was mentioned 619 times. This quickly tapered off, however, with only 39 mentions between 3 January and 6 May 2013.

Although the issue of interaction between grapefruit and medication was covered heavily by the major television news channels in November of 2012, the findings were not altogether new. As discussed above, the interaction between grapefruit and medication was initially found in the early 1990s [19, 22, 23]. Since then, many other scientists have studied the potential for interaction [24–29]. The impact of this interaction on sales was investigated by Lee and Brown [30], who estimated a double log demand model using the frequency of major newspaper headlines on grapefruit-medicine interactions to investigate the media effect on grapefruit juice sales. Even though they found a potential structural change due to the information, the newspaper hits did not significantly explain decreasing sales of grapefruit juice. The purpose of this paper is to measure the effect of health information on television and the internet on grapefruit and grapefruit juice consumption. Special attention is given to news relating to grapefruit-medicine interactions. Using the survey data, this study focuses on measuring the effect based on an individual’s perceived tone of the news release.

## Methods

### Survey design

An online survey was developed to understand respondents’ attitudes about health news on television and the internet and to measure the effect of media coverage on consumption changes of grapefruit and grapefruit juice. Tables 1 and 2 include detail descriptions of questions and basic statistics of variables. The first part of the survey determined exposure to grapefruit/grapefruit juice news media information of any type for the last month and attitudes toward health information in the media (television or internet news). Respondents were asked to indicate their level of disagreement/agreement for three questions using a seven-point Likert scale (1 equals strongly disagree, 4 equals neither agree nor disagree, and 7 equals strongly agree): 1) I trust health information I hear on television news or read on the internet news, 2) health information from television or internet news influences my consumption behavior, and 3) if I hear news health information from news sources (like the television or internet), I usually search for more information. These statements measure respondents’ actions in response to health information. The first statement is relatively passive in comparison to the second and third statements.

**Table 1:** Sample descriptive statistics and variable description

Variable (Dependent variable)		Variable description and code^a^	Sample (*N* = 3,504) %	U.S. Census %
Media expose (Y_1_)	=1 if	Respondents have been exposed to grapefruit/grapefruit juice news over the past month	15.8	NA
Gender	=1 if	Male	45.0	48.5^b^
Age	N	Mean = 46.1 (Standard deviation = 15.7), range = 18 to 76	-	-
Household income	=1 if	Under $25,000	28.1	25.7
	=2 if	$25,000 to $49,999	32.0	24.7
	=3 if	$50,000 to $74,999	19.4	17.7
	=4 if	$75,000 or more	20.6	31.9
Education	=1 if	Less than high school	2.6	12.9
	=2 if	High school and some college	63.0	57.2^c^
	=3 if	College and more	34.4	29.9^c^
Trust	=1 if	Respondents somewhat agree/agree/strongly agree that they trust health information they hear on TV news or read on internet news	55.5	NA
Influence	=1 if	Respondents somewhat agree/agree/strongly agree that health information from TV or internet news influences their consumption behavior	49.2	NA
Search	=1 if	Respondents somewhat agree/agree/strongly agree that if they hear new health information from news sources (like the TV or internet), they usually search for more information	60.4	NA
Yes-warning	=1 if	The medications come with warnings about grapefruit or grapefruit juice of respondents who take prescribed medication	14.7	NA
No-warning	=1 if	The medications come with no warnings (or unsure) about grapefruit or grapefruit juice of respondents who take prescribed medication	43.2	NA
Occasionally cons.	=1 if	less than once a month or once a month	45.7	NA
Frequently cons.	=1 if	2-3 times a month, once a week, 2–3 times a week or daily	17.9	NA
			Subsample (*N* = 552)^d^ %	
Consumption changes (Y_2_)	=0 if	Respondents consumed less than before	10.9	
	=1 if	Respondents did not change their consumption	72.3	
	=2 if	Respondents consume more than before	16.9	
Positive tone	=1 if	Respondents recalled the news was positive	35.5	
Negative tone	=1 if	Respondents recalled the news was negative	27.4	
Positive freq	N	Frequency of positive news (0 to 8), mean =1.5	-	
Negative freq	N	Frequency of negative news (0 to 8), mean =1.3	-	
Old news	=1 if	Respondents already knew of grapefruit-medicine interaction	40.4	
New news	=1 if	The news of grapefruit-medicine interaction was new for respondents	24.5	

^a^Alternative code for the dummy variables, media expose, gender, trust, influence, search, yes-warning, no-warning, low consumption, high consumption, positive tone, negative tone, old news and new news is ‘0’

^b^Age 18 years and over

^c^Completed four years of high school and more; four years of college or more

^d^The described percentages of subsample are only for respondents who were exposed to media coverage (i.e. media expose =1)

**Table 2:** Exposure rates of health news and perceived news tones

	Exposure rate (%)	Positive tone (%)	Negative tone (%)	Neutral or don’t recall (%)
Grapefruit interacting with medications	64.9	19.8	39.4	40.8
Grapefruit helps you lose weight	36.2	54.0	15.5	30.5
Drinking GFJ helps you to get vitamin C	33.6	54.3	11.3	34.4
Grapefruit diet	25.9	58.7	9.1	32.2
Grapefruit helps lower cholesterol levels	21.0	62.9	8.6	28.5
Grapefruit helps in cancer prevention	18.1	62.0	9.0	29.0
Grapefruit treats common ailments	14.8	67.1	11.0	22.0
Calories in grapefruit	14.1	65.4	6.4	28.2
Grapefruit seed extract	12.7	67.1	5.7	27.1
Grapefruit helps improve your skin	12.1	70.2	4.5	25.4
Grapefruit and higher blood level	11.2	66.1	11.3	22.6
Prevents arthritis and works as an antiseptic	10.5	72.4	8.6	19.0
Funny You-Tube video about GF/GFJ	5.2	89.7	3.5	6.9

Individuals’ attitudes were measured for how reliable they considered the health information, the influence of news on their consumption behavior, and their initiative on searching for further information related to the news. Consumers who considered the media information more reliable were thought to be more influenced by the news and more likely to search for further information. As a result, these individuals were expected to more likely be exposed to such health information. In addition, if respondents took any prescription medications, the individuals’ consumption of grapefruit or grapefruit juice and their knowledge about prescription medications were taken into consideration. The expectation was that consumers who took these medicines, or who consumed grapefruit/grapefruit juice, might be more likely to be aware (pay attention) to the news.

The second part of the questionnaire involved participants who were exposed to news about grapefruit/grapefruit juice in the last month. Those participants were asked to answer a series of questions about how they perceived the tone of the news, how many times they saw/heard the news, what types of news they saw/heard, whether or not the news was new information for the respondents, and how they have changed their consumption of grapefruit/grapefruit juice after seeing/hearing the news. Respondents who clearly recalled the tone of the news were assigned to either the positive (very positive or generally positive) news group or the negative (very negative or generally negative) news group. Individuals indicated the number of positive or negative news stories they saw/heard for the same periods. In addition, respondents were asked to categorize the news by choosing from a list containing the following choices: grapefruit-medicine interaction, diet, high blood pressure, vitamin C, calories, weight loss, cholesterol level, cancer and/or arthritis prevention, treatment of ailments, antiseptics, and skin improvement. A prompt question asked whether or not the news they selected contained new information. Finally, respondents were asked to indicate their consumption changes based on the news stories they had seen, read, or heard in the last month. In particular, this study identified consumers exposed to the grapefruit-medicine interaction news and made a distinction between consumers who had heard the news for the first time and consumers who had heard this information before.

Finally, all participants were asked to indicate their grapefruit/grapefruit juice consumption frequency and their socioeconomic status, age, gender, income, and education, as well as whether they take prescribed medications and whether they recognize that the medications were at risk of grapefruit-medicine interaction. The survey questionnaire was approved by the institutional review board (IRB) before data collection. The IRB found that this research study presents no more than minimal risk to participants.

### Modeling media exposure and consumption changes

Consumers not exposed to media information on grapefruit-medicine interaction are likely to be a source of bias in measuring the effect of media coverage on choice and behavior. If the percentage of non-exposure is significant, then the estimated total effect of media information may have a greater influence. Therefore, adequate measures of market response to media coverage depend on understanding the nature of individuals who are or are not exposed to the news media. Standard ordinary least square estimates under the non-randomly selected samples are inconsistent and biased. In other words, the estimated values do not represent the true values very well. Heckman [14] introduced self-selection bias and treated the unobserved selection factors as a problem of specification error or a problem of omitted variables. Therefore, Heckman used a two-step procedure to correct the bias in the estimation of the outcome equation by explicitly using information gained from the model of sample selection.

Unlike the standard Heckman approach, respondents’ consumption changes are observed with ordinal measurements such as consume more, unchanged, or consume less. The nature of the outcome value does not enable a straight-forward application of Heckman’s two-step procedure due to the non-linearity of the conditional mean in the second estimation step [31]. Instead, De Luca and Perotti [31] applied the idea of simple estimators by Lewbel [32] and proposed a maximum likelihood (ML) estimation incorporating binary selection and ordered responses. An ML estimator computes the contributions to the likelihood function for the possible outcomes.

In the study, we define *W* as those distinguishing respondent attitudes toward health information on media, such as reliability, influence, and initiative, and let *X* reflect individual health conditions and characteristics, such as age, gender, education, etc. We also let matrix *Z* include both *W* and *X*. Thereby, the selection equation can be expressed as

1 $$ {Y}_{1i}^{\ast }={Z}_i^{\prime}\gamma +{\mu}_i,\kern0.5em {Y}_{1i}=I\left({Y}_{1i}^{\ast}\ge 0\right) $$

where ${Y}_{1i}^{\ast }$ is the latent variable measuring the exposure rating underlying the propensity to be exposed to media coverage, and *Y*_1*i*_ is a dichotomous variable of exposure indicating whether or not individuals watched, read, and/or heard about grapefruit/grapefruit juice news over the last month. The function *I* has the value of 1 if the latent variable is greater than or equal to zero and has the value of 0 if the latent variable is less than zero.

Only the respondents who were exposed to the media coverage (i.e., *Y*_1*i*_ = 1) answered consumption change with consume less, no changes, or consume more. The outcome equation is

2 $$ {Y}_{2i}^{\ast }={X}_i^{\prime}\beta +{\varepsilon}_i,\kern0.5em {Y}_{2i}={\displaystyle {\sum}_{h=1}^Hh\cdot I\left({\alpha}_{h-1}<{Y}_{2i}^{\ast}\le {\alpha}_h\right)},\kern0.5em  if\;{Y}_{1i}=1 $$

where ${Y}_{2i}^{\ast }$ is the latent variable measuring consumption change ratings after exposure to a particular type of media, *Y*_2*i*_ is the indicated ordered response, and *α* = (*α*_0_, *α*_1_, …, *α*_*h*_) are the unknown cutoffs of the H-alternative ordered model with *α*_0_ = − ∞, *α*_*h*_ = + ∞, and *α*_*h* − 1_ < *α*_*h*_. The regression parameters *γ* and *β*, as well as the (*h* − 1) threshold values *α*_1_, …, *α*_*h* − 1_, are the parameters to be estimated. The thresholds are cut-points on the latent variable used to differentiate changing points given that all the predictor variables are set at zero. The sign of the regression parameter *γ* and *β* can be immediately interpreted as determining whether or not the latent variable ${Y}_{1i}^{\ast }$ or ${Y}_{2i}^{\ast }$ increases with the regressor. The joint distribution function of (*μ*_*i*_, *ε*_*i*_) is assumed to be Gaussian, with the zero means, unit variances, and correlation coefficient defined as *ρ*. The probabilities of the possible outcomes can be expressed as

3 $$ \begin{array}{l} \Pr \left({Y}_{1i}=0\right)=1-\Phi \left({Z}_i^{\prime}\gamma \right),\\ {} \Pr \left({Y}_{1i}=1,\ {Y}_{2i}=h\right)={\Phi}_2\left({Z}_i^{\prime}\gamma,\ {\alpha}_h-{X}_i^{\prime}\beta; - \uprho \right)-{\Phi}_2\left({Z}_i^{\prime}\gamma,\ {\alpha}_{h-1}-{X}_i^{\prime}\beta; - \uprho \right)\end{array} $$

The regression parameters *γ*, *β*, *α*, and *ρ* are obtained by maximizing the likelihood function,

4 $$ \mathrm{L}={\displaystyle \prod_{i=1}^n} \Pr {\left({Y}_{1i}=0\right)}^{1-{Y}_{1i}}{\displaystyle \prod_{h=1}^H} \Pr {\left({Y}_{1i}=1,\ {Y}_{2i}=h\right)}^{Y_{1i}I\left({Y}_{2i}=h\right)} $$

The model was estimated using the ‘OPSEL’ Stata command by De Luca and Perotti [31]. The OPSEL provides the parametric maximum likelihood (ML) estimator of an ordered responses model with sample selection. The errors are assumed to be a bivariate Gaussian distribution.

Marginal effects are useful information for comparing relative effectiveness across covariates on probability changes. In many studies, researchers calculate marginal effects at a given covariate point (usually at means) because the marginal effect in non-linear models is not constant over a range. When a non-linear model includes interaction terms, the marginal effect of a variable is varied by other interacting variables, and estimated coefficients of interaction terms do not provide the change in the partial effect of either variable (Buis [33], Karaca-Mandic, Norton and Dowd [34], Jaccard [35] and Greene[36]). Karaca-Mandic, Norton and Dowd [34] explained that the significance of interaction terms indicates improvement of the goodness of fit of the model, while it does not indicate a significance of a cross-partial effect, and the sign of interaction terms is not the sign of a cross-partial effect. Following Greene [36], we used graphic analysis to specify the change of probability by changing interesting covariates, in particular interaction terms. We focus on calculating expected values expressed as Equation 5, given specific characteristics of respondents to control all other covariates and draw graphs to show the effect.

5 $$ \begin{array}{l}E\left({Y}_{1i}=1\right)= \Pr \left({Y}_{1i}=1\Big|Z\right)=\varPhi \left({Z}_i^{\hbox{'}}\gamma \right)\\ {}E\left({Y}_{2i}=h\Big|{Y}_{1i}=1\right)=\frac{ \Pr \left({Y}_{1i}=1,{Y}_{2i}=h\right)}{ \Pr \left({Y}_{1i}=1\right)}\\ {}\kern6.75em =\frac{\varPhi_2\left({Z}_i^{\hbox{'}}\gamma, {\alpha}_h-{X}_i^{\hbox{'}}\beta; -\rho \right)-{\varPhi}_2\left({Z}_i^{\hbox{'}}\gamma, {\alpha}_{h-1}-{X}_i^{\hbox{'}}\beta; -\rho \right)}{\varPhi \left({Z}_i^{\hbox{'}}\gamma \right)}\end{array} $$

## Results

### Sample characteristics

The data for measuring the effect of media coverage on grapefruit/grapefruit consumption changes were collected using an online survey between 10 and 17 December 2012, approximately two weeks after the news about the grapefruit-medication interaction was released. Panelists were recruited by Toluna® via web banners, public relations, website referrals, and other methods. Toluna® (Toluna USA, Dallas, Texas) controls panel quality using *GeoIP* and postal codes, double opt-in procedures, and internet cookies to prevent duplication, and imposes an age restriction of eighteen years of age or older. Initially, 3,921 respondents started the survey. Of those, 3,504 respondents consented to participate in the survey, were adults, and passed a validation question which ensures that respondents were carefully reading each question.

Variable definitions and basic descriptions of respondent characteristics are shown in Table 1. Descriptive statistics for variables that are only associated with respondents who have been exposed to grapefruit news are summarized in the lower level of Table 1. Demographics of gender, age, household income, and education for adults from the U.S. census are provided for comparison to the sample. The respondents in the sample were slightly weighted to educated and female. Of the total participants, approximately 64 % of the respondents consumed grapefruit or grapefruit juice at least occasionally. Of those, 72 % (46 % of the total) of the respondents consumed grapefruit or grapefruit juice less than once a month or once a month, and 28 % (18 % of total) consumed grapefruit or grapefruit juice at least 2–3 times a month. Approximately 58 % (15 % of ‘Yes-warning’ and 43 % of ‘No-warning’) of the total participants indicated that they take a prescription medication. Of those, 25 % (15 % of total) were aware that their medications come with warnings about grapefruit or grapefruit juice interactions, while 75 % (43 % of total) were unaware or unsure about the grapefruit-medicine interactions. Major sources (over 80 %) of the warnings of the medication-grapefruit interaction were doctor (60 %), nurse/nurse practitioners (14 %) or pharmacists (53 %). In addition, our data showed that 50.3 % of respondents aged 20–59 took prescription medications and 83.1 % of respondents (aged 60 and over) took prescription medications.

In addition to individual socio-economic characteristics, it is important to understand how consumers react to health information on television and the internet. Approximately 56 % of the respondents at least somewhat trusted health news obtained from television and the internet while about 49 % of the respondents indicated that the news media influenced their consumption behavior (see Table 1). Interestingly, approximately 60 % of the respondents indicated that they searched for more information about the news information.

Of the total respondents, approximately 16 % indicated that they have seen, read, or heard of the grapefruit/grapefruit juice news in the last month. Of those who had heard the news, approximately 36 % and 27 % of the respondents recalled that the news was either positive or negative in tone, respectively. On average, they heard positive news 1.5 times and negative news 1.3 times. Approximately 65 % (Old news and New news) of the subsample indicated that one of news that they have seen, read or heard about grapefruit was related to grapefruit interaction with medication. In addition, approximately 25 % indicated that the grapefruit-medicine interaction was brand new information to them, and approximately 40 % already knew the information.

A summary of the health news to which the respondents had been exposed is shown in Table 2. Approximately 65 % of the respondents who have seen, read, or heard about grapefruit/grapefruit juice indicated that one of the news stories was about grapefruit-medicine interaction, followed by news about grapefruit and weight control (36 %) and news about vitamin C (34 %). Based on the respondents’ perception of the tone of the news, we looked into the percentages of participants who perceived the tone of the news as negative or positive. Approximately 40 % indicated that the tone of the grapefruit-medicine interaction news was negative, 20 % recalled the news as positive, and 40 % did not recall the tone. The various responses to the news information may be due to the degree of consumer knowledge about the news information. Slooten, Friedman, and Tanner [37] found that individuals had trouble understanding health news due to limited health terminology literacy.

### Empirical models

Empirical models in this study are specified below by selection and outcome equations. The empirical model will discover not only the main effect of covariates, but the interaction effect of age and its relevant covariates on probability changes. Generally, consumer age links to the likelihood of taking prescription medications and grapefruit consumption. Approximately 45 % of adults aged 60 and over took cholesterol-lowering medicines [38]. Medication for high blood pressure and cholesterol-lowering medicines are often of the category of medications that interact with grapefruit. Considering the nature of the relationship between age and prescription medication/grapefruit consumption, the model includes four interaction terms.

$$ \begin{array}{l}\mathrm{Covariates}\ \mathrm{in}\ \mathrm{selection}\ \mathrm{equation}:\ \left\{\mathrm{Trust},\ \mathrm{Influence},\ \mathrm{Search},\ Xs\right\}\\ {}\mathrm{Covariates}\ \mathrm{in}\ \mathrm{outcome}\ \mathrm{equation}:\ \Big\{\mathrm{Positve}\ \mathrm{tone},\ \mathrm{Nagative}\ \mathrm{tone},\ \mathrm{Positive}\ \mathrm{frequency},\ \\ {}\kern13.25em \mathrm{Negative}\ \mathrm{frequency},\ \mathrm{Old}\ \mathrm{news},\ \mathrm{New}\ \mathrm{news},\ Xs\Big\}\\ {}Xs=\Big\{\mathrm{Age},\ \mathrm{Gender},\ \mathrm{Income},\ \mathrm{Education},\ \mathrm{Yes}\ \mathrm{warning},\ \mathrm{No}\ \mathrm{warning},\ \mathrm{Occasional}\ \mathrm{consumption},\ \\ {}\kern2.25em \mathrm{Frequent}\ \mathrm{consumption},\ \mathrm{Age}\times \left(\mathrm{Yes}\ \mathrm{warning},\ \mathrm{No}\ \mathrm{warning},\ \mathrm{Low}\ \mathrm{consumption},\ \mathrm{High}\ \mathrm{consumption}\right)\Big\}\end{array} $$

### Estimated results of media exposure and consumption changes

Only about 16 % of the total respondents indicated that they had watched, read, and/or heard about grapefruit/grapefruit juice in the news for the last month. Because the percentage of respondents who did not indicate news exposure was significant (84 %), it was important to use an adequate model to account for both sample selection and ordered responses. In this case, we used a binary probit for media exposure (selection equation) and an ordered probit for changes in grapefruit/grapefruit juice consumption (outcome equation). Equation 4 was estimated using maximum likelihood estimation and the estimated results are summarized in Table 3. Note that before running the model, we checked the internal consistency and reliability of three statements, Trust, Influence and Search, whether the set of variables measures a single item. A Cronbach’s alpha statistic of 0.7 or higher is considered acceptable to measure correlation [39]. The estimated Cronbach’s alpha was 0.75, indicating that the three statements measure the same construct of consumers’ attitude toward health news on television and the internet.

**Table 3:** Maximum likelihood estimation of sample selection and ordered responses

	Outcome equation (Y2^1)^)		Selection equation (Y1^2)^)	
	Estimated parameters	Standard error	Estimated parameters	Standard error
Thresholds 1	0.059	(1.348)	−2.550^b^	(0.242)
Thresholds 2	3.229^b^	(1.059)	-	
Age	0.020^a^	(0.011)	0.013^b^	(0.004)
Gender	0.103	(0.125)	−0.029	(0.056)
Income	−0.046	(0.059)	0.012	(0.026)
Education	0.174	(0.124)	0.117^b^	(0.056)
Yes warning	1.505^b^	(0.566)	0.798^b^	(0.314)
No warning	0.273	(0.396)	0.208	(0.186)
Occasionally consumed	1.646^b^	(0.539)	0.780^b^	(0.214)
Frequently consumed	2.150^b^	(0.669)	1.754^b^	(0.24)
Trust	-		0.012	(0.066)
Influence	-		0.173^b^	(0.067)
Search	-		0.257^b^	(0.063)
Positive tone	0.845^b^	(0.177)	-	
Negative tone	−0.277^a^	(0.167)	-	
Positive frequency	0.050	(0.038)	-	
Negative frequency	−0.038	(0.038)	-	
Old news	−0.398^b^	(0.165)	-	
New news	−0.505^b^	(0.181)	-	
Age*Yes warning	−0.037^b^	(0.011)	−0.006	(0.006)
Age*No warning	−0.007	(0.009)	−0.003	(0.004)
Age*Occasionally consumed	−0.035^b^	(0.010)	−0.011^b^	(0.004)
Age*Frequently consumed	−0.030^b^	(0.011)	−0.022^b^	(0.005)
*ρ*	0.278	(0.457)		
LR test (*H*_*o*_ : *ρ* = 0), *χ* ^2^ (1)		270.99^b^		
Observations		3497		
Log Likelihood		−1700.55		
Wald test, *χ* ^2^ (18)		169.51		
Estimated probabilities		Mean	Min	Max
p(y1 = 1)		0.157	0.016	0.654
p(y1 = 1, y2 = 0)		0.011	0.000	0.247
p(y1 = 1, y2 = 1)		0.127	0.134	0.455
p(y1 = 1, y2 = 2)		0.019	0.000	0.496

^b^ and ^a^ indicates that estimated parameters are significantly different from zero at 5 % and 10 % levels, respectively

1) Y2 = 0 if respondents consumed less than before, Y2 = 1 if respondents did not change their consumption and Y2 = 2 if respondents consume more than before

2) Y1 = 1 if respondents have been exposed to grapefruit/grapefruit juice news over the past month

A likelihood ratio (LR) test rejected the null hypothesis of independent equations (i.e., the estimated correlation coefficient, *ρ* is equal to zero). The correlation coefficient between the errors of outcome and the selection equations was positive for the results of the total sample (*ρ* = 0.278). If the correlation coefficient is positive, the unobserved factors that increase the probability that a respondent is exposed to grapefruit/grapefruit juice news are positively correlated with unobserved factors that increase grapefruit/grapefruit juice consumption. Also, a likelihood ratio test was conducted to compare the goodness of fit of the model with/without interaction terms (LR = −2×(−1720.79-(−1700.55) = 40.48, χ^2^(4) = 40.48). The test result rejected the null hypothesis that the two models fit equally well, which indicated that the model with interaction terms provides a better fit. To indicate the interactions effect, we picked a particular demographic group as baseline, this group being defined as male, household income between $50 K and $75 K, obtained college degree, non-grapefruit consumer, not taking prescription medication, unsure whether a respondent has received the information of grapefruit-medication interactions before, trust health news from television and the internet, influenced by the news and searching for more information about the news.

First, we focused on the results from the selection equation. The estimated results showed that consumers who indicated that health news on television or the internet impacted their consumption behavior and that they searched for more information tended to be more aware of news about grapefruit. In particular, the partial effect of consumers who indicated that they searched for more information (0.26) was greater than the partial effect of consumers who indicated that the health news release influenced their consumption behavior (0.17). Even though the awareness of the grapefruit news release was positively related with consumers’ trust of health news on television or the internet, the effect was not significant.

**Fig. 1:**
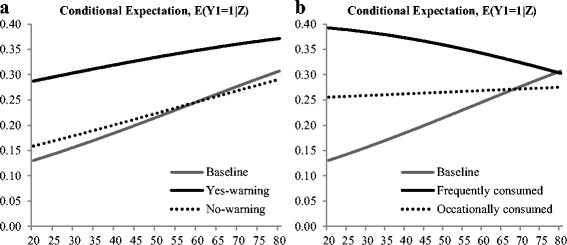
Conditional probability of awareness of grapefruit news releases for a particular demographic group, **a** changes by prescription medication status over age and **b** changes by frequency of grapefruit consumption over age

Of the socio-economic characteristics, age and education significantly explained the awareness of the grapefruit news release. Educated consumers were significantly more likely to have been exposed to the grapefruit news release, while respondents’ gender and income were not significant. Figure 1a shows the effect of interaction terms between age and status of prescription medications. Overall, as age increases, respondents are more likely to be aware of the grapefruit news releases. However, the probability of awareness is significantly higher for respondents who take prescription medication when the medication has an interaction effect with grapefruit consumption (Yes-warning) compared to respondents who do not take any prescription medication (Baseline) or who take prescription medication but the medication does not have an interaction effect with grapefruit consumption (No-warning). The probability between baseline and No-warning is similar as age increases. Figure 1b shows the interaction effects between age and levels of grapefruit consumption. The awareness of the grapefruit news release is significantly different between grapefruit consumers (Frequent and Occasional consumers) and non-consumers (Baseline). The probability of awareness increases as non-consumers’ ages increase, while the probability decreases as frequent consumers’ ages increase. Overall, the probability is stable for consumers who occasionally consume grapefruit as said consumer age.

To better understand respondents’ response to released grapefruit news, consumers who were exposed to the grapefruit news release indicated that their grapefruit/grapefruit juice consumption changes were based on the news they have seen, read or heard. The results are presented under the estimated results of the outcome equation in Table 3. Demographic variables except for age did not significantly influence grapefruit/grapefruit juice consumption changes of the respondents who are aware of the grapefruit news release.

The perceived tone of the health news release had a significantly larger relationship with consumption changes compared to frequency of exposure to media health news. Consumers whose general impression of the news stories about grapefruit consumption was positive significantly increased their consumption, and negative tones in the health news stories are significantly less likely to increase consumption compared to consumers who are neutral or don’t recall the tones. The parameter scale for news stories with a positive tone is greater than the parameter scale for those with a negative tone.

**Fig. 2:**
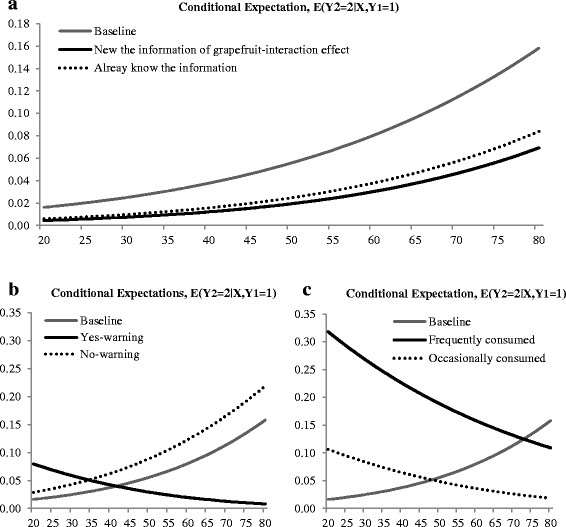
Conditional probability of increasing consumption for a particular demographic group given respondents exposed grapefruit news: **a** awareness of grapefruit-medication interactions, **b** changes by prescribed medication status over age, **c** changes by frequency of grapefruit consumption over age

Both the consumers who already knew the effect of grapefruit-medicine interaction and those hearing the news release for the first time indicated a lower likelihood to increase their consumption of grapefruit and/or grapefruit juice as shown in Fig. 2a. The probability of consumers being exposed to the health news for the first time was smaller, which indicates that respondents who already knew the news information may be less likely to perceive the news as negative, although it still influences grapefruit consumption changes. The slope of the tangent line is steeper as consumers’ ages increase. That is, older respondents are more subject to be influenced by the information.

Figure 2b shows the interaction effect of age with status of prescription medication. Consumers who do not take prescription medications or who take prescription medication but said the medication does not have an interaction effect with grapefruit consumption are more likely to increase consumption of grapefruit and/or grapefruit juice as age increases. However, respondents who are aware of grapefruit-mediation interactions for their prescribed medication are less likely to increase grapefruit consumption as age increases. Also, the probability of increasing grapefruit consumption switches at age 42, from which point, the probability of respondents who take prescription medication and the medication has warnings against consuming grapefruit is lower than respondents who do not take prescription medication.

Figure 2c shows the interaction effect of age with levels of grapefruit consumption frequency. The probability of increased grapefruit consumption increases as non-consumers’ (Baseline) ages increase, while the probability decreases as grapefruit consumers’ (frequently consumed and occasionally consumed) ages increase. The probability of frequent grapefruit consumers is higher than occasional consumers over the age ranges. Compared to non-consumers, frequent consumers have a high probability of increased grapefruit consumption until age 72 and occasional consumers have a high probability until age 48.

The estimated probabilities were calculated Table 3. Overall, the probability of consumers being recently exposed to the grapefruit health news was 16 %. Therefore, approximately 16 % of the adults heard/saw the grapefruit health news over the month. Of consumers who heard/saw the grapefruit news coverage, the majority (81 %) indicated that the news did not influence their grapefruit/grapefruit juice consumption, while approximately 19 % were influenced. Overall, only about 1.1 % of consumers decreased their grapefruit consumption, while 1.9 % of consumers increased given the time of grapefruit news release.

## Discussion

A national survey by the National Health and Nutrition Examination Survey (NHNES) 2007/08, Center for Disease Control and Prevention (CDC)/ National Center for Health Statistics (NCHS) [38], indicated that 48.3 % and 88.4 % of Americans used at least one prescription medication in the 20 to 59 age group (aged 20–59) and the 60 years old and over age group (aged 60 and over), respectively. We used the 2007 U.S. population information to approximate the number of people taking prescription medications within the different age groups. Of the Americans aged 20 years old and over, 57.6 % were predicted to take prescription medications. This statistic is comparable to our findings that 58 % of respondents indicated that they take prescription medication.

Consumers are exposed to a tremendous amount of information. Development of mobile devices has enhanced the speed of dissemination of news and increased opportunities to receive news. Kalaitzandonakes, Marks and Vickner [40] found consumers’ perception and/or behaviors are influenced by information from media coverage. The number of internet hits was an important indicator to measure exposure rates. However, it is physically impossible to count internet hits exactly because sources of information are ever growing. Respondent attitudes toward news or information would be potential indicators to measure media effect instead of internet hits. In this study, we found that consumers’ attitudes toward health news on television and the internet significantly explained the selectivity of exposure to grapefruit news using three different reactions: reliability of the news, effects on consumption behavior, and initiative to search for further information related to the news. Generally, relatively aggressive consumers (i.e., searching for more information) tended to have more exposure to the grapefruit news release, which implies that these consumers were willing to search for new information rather than wait for the news to come to them. This result is consistent with Freedman and Goldstein [41] who found that television viewing time and total negative ads aired did not significantly influence voter turnout, while the tone of the ads and individuals’ tendencies toward the ads significantly influenced voter turnout.

The study results confirm the important role of media releases in consumer perception and food consumption. News stories with positive tones lead to increased consumption, while news stories with negative tones lead to decreased consumption. The citrus industry regularly promotes the benefits of consuming grapefruit/grapefruit juice. Zheng and Kaiser [42] indicated that if the market for a commodity is saturated, then advertising’s ability to enhance demand will be attenuated due to the limited consumption potential for the good. This frequently means that continuous advertising does not increase sales, but when advertising stops, sales may drop. Active engagement by the citrus industry on regularly promoting the benefits of consuming grapefruit/grapefruit juice would seem to be an advantageous marketing strategy. In particular, marketing activity aimed at conveying a positive perception may offset negative reactions to warnings about grapefruit consumption.

In addition, health information that is repeated through media influences both consumers who already know and those who are hearing about it for the first time. That is, whenever the issue receives media coverage, grapefruit consumption is influenced. Smith et al. [43] suggested in their study of sun protection behavior and media information that since the impact of mass media on improving sun protection behavior is not sustained, repeated and supplemented strategies are necessary. Therefore, repeated media releases remind consumers of the interaction effect and lead to less consumption of grapefruit. This finding may apply to public education. That is, repeated public education about healthy eating and nutritional information may gradually influence consumers’ perception and food choices.

Respondents more strongly react to media information when the information is directly related to their health. Our findings demonstrated that consumers who take prescription medication with warnings about grapefruit consumption reacted strongly. Unfortunately, the major demographics of grapefruit consumers are frequently the same people most likely taking medication that interacts with grapefruit [13]. Therefore, it is important to provide correct information not only to the consumers who take the medication, to reduce their likelihood of experiencing side effects, but to the other consumers in order not to discourage them from consuming grapefruit.

Grapefruit consumers who occasionally or frequently consumed grapefruit/grapefruit juice still have a high probability to increase grapefruit consumption changes after watching the news release about grapefruit, but this group of consumers becomes more vulnerable to the grapefruit health news as age increases. Negative health news such as the effect of grapefruit-medicine interactions bothers grapefruit consumers. The probability of increased grapefruit consumption decreases quickly for occasional consumers (the probability at age 80 was decreased by 83 % compared to the probability at age 20) compared to frequent consumers (at the same age differences, the probability decreased by 67 %) as age increases. Loyal consumers may be well aware of the effect and may know how to handle the problem.

According to the Florida Department of Citrus, total grapefruit juice sales were 1,699 thousand gallons in December of 2011 and 1,607 thousand gallons in December of 2012. Although there was an approximate decrease of 5 % for grapefruit juice sales in December of 2012 compared to the previous year, when we consider decreasing trends of grapefruit juice sales (on average, grapefruit juice sales have declined by 5–6 % annually since 2010) and price increases (grapefruit juice prices in the season of 2012/13 increased by 3.9 % compared to the prior season of 2011/12), the reduction of grapefruit juice sales may not be related to the news release. This result may be consistent with the finding of Lee and Brown [30]. However, as found in the study, the tones perceived by consumers and repeated information will eventually influence food consumption, over the long term.

## Conclusions

Although the relationship between grapefruit and medication was first reported in the early 1990s, news about this relationship was released again in November of 2012. A consumer survey was conducted two weeks after the news was covered by national broadcasters in 2012. Approximately 16 % of the survey participants indicated that they had heard news about grapefruit/grapefruit juice during the month prior to the survey, when the story was being reported. A sample selection model was estimated for accurate measurement of media effect based on understanding of the characteristics of consumers exposed to news media. In addition, we used the individuals’ own perception of the tone of the news to better measure the effect.

Results show that exposure to media information on grapefruit-medicine interaction does have a tendency to result in reduced consumption; however, only a small proportion of consumers are both exposed to such media and are active consumers of grapefruit/grapefruit juice. In particular, the effect varied by consumer’s age, grapefruit consumption frequency and conditions of taking medication that may interact with grapefruit consumption. Although consumer’s age was positively related to the probability of increased grapefruit consumption, when these consumers were taking the medication or were frequently consumed grapefruit, consumer’s age was negatively related to the probability of increased grapefruit consumption.

This study did not compare exact adjustments of grapefruit/grapefruit juice consumption based on the media information because measurements of consumption changes were self-reported as increased, decreased, or no change (magnitude of change was not collected). Despite this drawback, this study provides insight into the importance of consumer attitudes to media exposure and the importance of the perceived tone of the news on immediate reactions. Also, this study demonstrated that consumers react strongly to the news when the news is directly linked to their well-being.

## Abbreviations

CDC: Center for Disease Control and Prevention
NCHS: National Center for Health Statistics
NHNES: National Health and Nutrition Examination Survey
